# Measuring disease activity in COPD: is clinically important deterioration the answer?

**DOI:** 10.1186/s12931-020-01387-z

**Published:** 2020-06-02

**Authors:** Dave Singh, Gerard J. Criner, Ian Naya, Paul W. Jones, Lee Tombs, David A. Lipson, MeiLan K. Han

**Affiliations:** 1 University of Manchester, Medicines Evaluation Unit, Manchester University NHS Foundation Trust, Manchester, UK; 2 Lewis Katz School of Medicine, Temple University, Philadelphia, PA USA; 3 GSK, Respiratory Medicines Development Centre, Stockley Park, Middlesex, UK; 4 RAMAX Ltd, Bramhall, Cheshire, UK; 5 Precise Approach Ltd, London, UK; 6 GSK, Respiratory Clinical Sciences, Collegeville, PA USA; 7 Division of Pulmonary, Allergy, and Critical Care, Perelman School of Medicine, University of Pennsylvania, Philadelphia, PA USA; 8 Division of Pulmonary and Critical Care, University of Michigan Health System, Ann Arbor, MI USA

**Keywords:** CID, Clinically important deterioration, COPD, Deterioration, Health status, Long-term outcome, Lung function

## Abstract

Given the heterogeneity of chronic obstructive pulmonary disease (COPD), personalized clinical management is key to optimizing patient outcomes. Important treatment goals include minimizing disease activity and preventing disease progression; however, quantification of these components remains a challenge. Growing evidence suggests that decline over time in forced expiratory volume in 1 s (FEV_1_), traditionally the key marker of disease progression, may not be sufficient to fully determine deterioration across COPD populations. In addition, there is a lack of evidence showing that currently available multidimensional COPD indexes improve clinical decision-making, treatment, or patient outcomes. The composite clinically important deterioration (CID) endpoint was developed to assess disease worsening by detecting early deteriorations in lung function (measured by FEV_1_), health status (assessed by the St George’s Respiratory Questionnaire), and the presence of exacerbations. Post hoc and prospective analyses of clinical trial data have confirmed that the multidimensional composite CID endpoint better predicts poorer medium-term outcomes compared with any single CID component alone, and that it can demonstrate differences in treatment efficacy in short-term trials. Given the widely acknowledged need for an individualized holistic approach to COPD management, monitoring short-term CID has the potential to facilitate early identification of suboptimal treatment responses and patients at risk of increased disease progression. CID monitoring may lead to better-informed clinical management decisions and potentially improved prognosis.

## Background

Chronic obstructive pulmonary disease (COPD) is a complex and heterogenous condition with many components to its clinical presentation, including dyspnea, cough (with or without sputum), airflow limitation, reduced exercise capacity/fatigue, weight loss and exacerbations [1, 2]. As these components vary in both their presence and severity [1], personalization of the assessment and clinical management of COPD is key to optimizing patient outcomes, and is a proposed treatment approach according to the Global Initiative for Chronic Obstructive Lung Disease (GOLD) report [2].

**Fig. 1:**
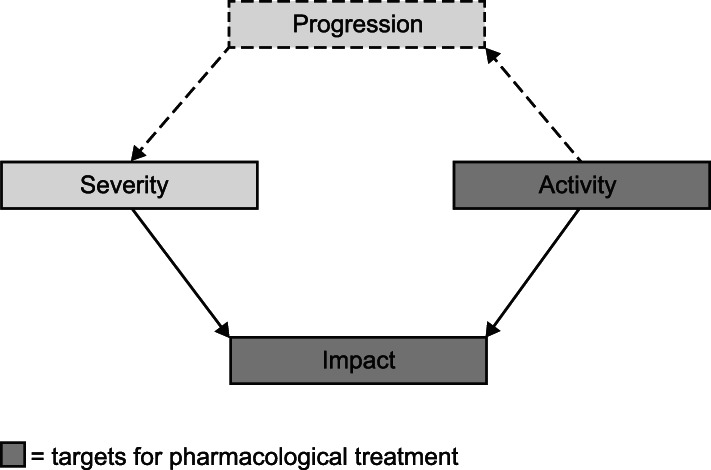
The relationships between the key components of COPD [4]. The relationships between components of COPD. Severity, (disease) activity, and impact are components of COPD; severity and activity determine the level of impact on a patient. Disease activity drives disease progression, which worsens severity. COPD, chronic obstructive pulmonary disease. Reprinted with permission of the American Thoracic Society. Copyright© 2019 American Thoracic Society. The American Journal of Respiratory and Critical Care Medicine is an official journal of the American Thoracic Society

The key features of COPD fall into three categories [3, 4]: (i) disease severity, which is the degree of functional impairment such as airflow limitation, hyperinflation and reduced exercise capacity; (ii) disease activity, such as exacerbations; and (iii) disease impact, which considers the patient’s perception of their disease. These aspects of COPD are intrinsically linked, with disease activity driving disease progression, which in turn worsens disease severity and increases the impact on the patient (Fig. 1) [4].

The GOLD report describes key treatment goals for the pharmacological management of stable COPD, which include reducing the risk of future COPD exacerbations, thereby minimizing disease activity and preventing disease progression [2]. The ultimate aim of this treatment approach is to reduce the level of disease impact on the patient. However, in both clinical studies and clinical practice, the quantification of disease activity and progression remains a challenge. This is due to both the lack of a clear definition to describe these terms, and a lack of clarity on how best to monitor disease progression.

There is a widely acknowledged need for a more comprehensive approach to the management of COPD, a disease in which deterioration may take different forms. This has led to the development of the composite clinically important deterioration (CID) endpoint to measure short-term disease worsening. This endpoint was first introduced in 2016 to test the benefits of bronchodilation [5]. Subsequently, numerous studies have used it to measure the efficacy of pharmacological interventions (including bronchodilators and inhaled corticosteroids [ICS]), albeit with some variation in the definitions used. This review article evaluates the evidence for the utilization of the CID endpoint in measuring disease activity in COPD.

### The rationale for a new approach to examine disease progression in COPD

The decline over time in forced expiratory volume in 1 s (FEV_1_) has traditionally been the key marker of disease progression [6]. Current regulatory guidance highlights longitudinal changes in FEV_1_ as a preferred primary outcome for clinical trials designed to assess the impact of pharmacological interventions on disease progression in patients with COPD [7]. However, meaningful deteriorations in health status can occur that are only weakly correlated with reductions in lung function [8]. Likewise, in a post hoc analysis of the 12-week CRYSTAL study, agreement between FEV_1_ responder status and clinically meaningful improvements in patient-reported outcomes (PROs) was only observed for a minority of patients with COPD. Additionally, an examination of responders using different health status and symptom questionnaires indicated little overlap between measures, highlighting that PROs are not interchangeable [9]. Thus it is widely acknowledged that COPD is a multi-faceted disease, the progression of which can be monitored in a number of ways, including the rate of decline in health status and other PROs, the change in exercise capacity or physical activity over time, and COPD exacerbation frequency over time [6]. As such, one dimension is unlikely to adequately capture all aspects of disease worsening [10].

The need for additional markers of disease progression beyond FEV_1_ is supported by evidence that a significant proportion of patients with COPD diagnosed using spirometry display stable or even improved FEV_1_ over time [11], although such patients may have experienced high rates of deterioration in the past. For example, in the 3-year observational cohort ECLIPSE study [12], 38% of patients displayed a mean annual rate of FEV_1_ decline > 40 mL, while 31% displayed either no significant change (±20 mL/year) or an improvement in FEV_1_ (> 20 mL/year) [11]. Similarly, another longitudinal cohort study showed that 54–58% of patients with COPD displayed FEV_1_ decline of < 40 mL/year [13].

There are several potential explanations for low rates of lung function deterioration in patients with COPD. In some, incomplete maturation of the lungs can result in reduced lung volumes but relatively stable lung function over time. For example, an analysis that combined three independent cohorts showed that ~ 50% of patients with COPD entered adult life with reduced FEV_1_ and met the spirometric criteria for COPD in middle age despite a relatively normal rate of FEV_1_ decline of ~ 25–30 mL/year [14]. There is also some evidence to suggest that patients categorized as having mild COPD (GOLD grade 1) experience more rapid deterioration in lung function than patients with GOLD grade 2 or 3 (FEV_1_ decline of 57 mL/year versus 43 mL or 31 mL/year, respectively, in patients aged ≤ 55 years, and 80 mL/year versus 53 mL or 28 mL/year in patients aged ≥ 65 years) [13]. These findings are in line with other studies showing that rapid declines in lung function can occur in the early stages of COPD [15]. In addition, high levels of dropout in long-term randomized controlled COPD trials, particularly among patients receiving less effective therapy, may lead to confounding and bias, which can contribute to an under-assessment of mean annual FEV_1_ decline or exacerbation frequency [16].

Alternatives to the use of a single measure such as FEV_1_ have been proposed, since the available evidence suggests that no single aspect of COPD is sufficient to adequately monitor disease progression [2, 8, 10, 17–20]. Several multidimensional COPD indexes have been developed that attempt to quantify disease activity and determine prognosis; however, although linked, disease progression and disease prognosis are not interchangeable. Examples of these prognostic indices include BODE (body-mass index, airflow obstruction, dyspnea, and exercise) [21], ADO (age, dyspnea, obstruction) [22], HADO (health, activity, dyspnea, obstruction) [22], CART (classification and regression tree) [23], DOSE (dyspnea, obstruction, smoking, exacerbation) [24] and SAFE (St George’s Respiratory Questionnaire [SGRQ] score, airflow limitation and exercise tolerance) [25]. However, these indices may be difficult to use in clinical studies and/or routine clinical practice, can have limited sensitivity or applicability to general COPD populations (since certain indices are used only in severe COPD [BODE] or elderly patients [ADO] [26]), or are restricted to one outcome (e.g. mortality). Furthermore, few of these indices have been validated in subsequent studies, with BODE being the notable exception [10, 17]. As a result, there is limited evidence that these prognostic indices can help improve decision-making, treatment or outcomes [26]. Therefore, as they have primarily focused on disease prognosis and have largely overlooked disease activity and the potential for disease progression, a new approach is needed to enable the reliable monitoring of disease activity and progression, which can be implemented in both clinical trials and routine clinical practice.

### The clinically important deterioration concept

The composite CID endpoint concept was developed to meet the need, highlighted in 2007 by Mahler and Criner [27], for a measure to assess the proportion of patients showing disease stability or worsening (which may be a marker of suboptimal treatment response or treatment failure) in response to pharmacological treatment. Development of the composite CID endpoint was based on following key principles: (i) it needed to address different aspects of disease progression; (ii) those components had to be largely independent of each other; (iii) it had to be better able to quantify future risk in individual patients than the assessment of individual components; and (iv) it should be able to discriminate between pharmacological therapies in short-term studies (≤ 6 months) in different disease subgroups.

Given these principles, suitable outcome measures and appropriate thresholds for deterioration of these measures were determined. Deterioration of lung function and exacerbations are well-established indicators of poor long-term prognosis in COPD [28–33]. For lung function, FEV_1_ was chosen, because it is a standard measure of efficacy used in trials of maintenance therapies. The selected threshold for deterioration was the minimum clinically important difference (MCID) of 100 mL change from baseline [34]. For exacerbations, the occurrence of a moderate (requiring treatment with oral corticosteroids and/or antibiotics) or severe (requiring hospitalization or an emergency room visit) exacerbation was included [2, 28]. For health status, SGRQ was selected since it is widely used in COPD trials and has an established MCID (≥ 4 units) [35]. In addition, SGRQ worsening has also demonstrated prognostic value, with results from the ECLIPSE study showing that worsening health status over the first year was associated with an increased risk of hospitalization and mortality over the following 2 years compared with patients who had an improvement in SGRQ score or no change [36].

Several recent, mainly retrospective, analyses have examined treatment impact on CID incidence [5, 37–45]. Most have used the three-component CID definition as described above, although other definitions have been explored (Table 1), which have included the COPD Assessment test (CAT) score, as a replacement for SGRQ, and the Transition Dyspnea Index (TDI).

**Table 1:** Efficacy trials examining the effects of treatment escalation on overall CID incidence

Study description	CID definition	Treatments	Patient population
**Dual bronchodilator combination therapy**			
First retrospective CID analysis of two 24-week double-blind trials [5]	FEV_1_, SGRQ, exacerbations	UMEC/VI vs placebo, TIO, UMEC or VI	High symptoms, mMRC score ≥ 2, low exacerbation risk
Retrospective pooled data from three 6-month double-blind trials [41]	FEV_1_, SGRQ, exacerbations	UMEC/VI vs TIO	High symptoms, mMRC score ≥ 2, low exacerbation risk. Analyses of the ITT population and maintenance-naïve subgroup (31% of patients)
Retrospective pooled analysis of three 26-week, randomized, double-blind trials (SHINE, LANTERN & ILLUMINATE) [38]	Definition 1: FEV_1_, SGRQ, exacerbations; Definition 2: TDI, SGRQ, exacerbations	IND/GLY vs SFC or TIO	High symptoms, low exacerbation risk (SHINE & LANTERN), exacerbation-free (ILLUMINATE)
Retrospective pooled analysis of two 24-week, randomized double-blind trials (AUGMENT & ACLIFORM) [44]	FEV_1_, SGRQ, TDI, exacerbations	ACL/FORM vs ACL, FORM or placebo	Low to high symptoms, low exacerbation risk
Retrospective 52-week randomized double-blind trial (FLAME) [37]	FEV_1_, SGRQ, exacerbations	IND/GLY vs SFC	High Symptoms, mMRC score ≥ 2, ≥ 1 exacerbation, stable on LAMA for 1 month
Retrospective 12-week, randomized, open-label, switching trial (CRYSTAL) [40]	Definition 1: FEV_1_, TDI, exacerbations; Definition 2: FEV_1_, CCQ, exacerbations; Definition 3: FEV_1_, CCQ, TDI, exacerbations	Switch to IND/GLY from previous ICS/LABA or a single LABA or LAMA	Low to high symptoms, low exacerbation risk on open-label therapy, mMRC score ≥ 1
Prospective 24-week, randomized, double-blind trial (EMAX) [46]	Definition 1: FEV_1_, SGRQ, exacerbations; Definition 2: FEV_1_, CAT, exacerbations; Definition 3: SGRQ, CAT, TDI, exacerbations	UMEC/VI vs UMEC or SAL	High symptoms, ICS-free population, ≤ 1 moderate exacerbation in the past year
**Multiple inhaler or single inhaler triple therapy**			
Retrospective pooled analysis of four 12-week, randomized double-blind trials [43]	FEV_1_, SGRQ, exacerbations	UMEC vs placebo added to existing open label ICS/LABA therapy	High symptoms, mMRC score ≥ 2, with or without exacerbations
Prospective, 52-week, randomized double-blind trial (FULFIL) assessed over 24 weeks (ITT population) and 52 weeks (extension population) [42]	Definition 1: FEV_1_, SGRQ, exacerbations; Definition 2. FEV_1,_ CAT, exacerbations	FF/UMEC/VI vs BUD/FORM	High symptoms, FEV_1_ < 50% and CAT ≥ 10 or FEV_1_ ≥ 50 to < 80% and CAT ≥ 10, and ≥ 2 moderate or ≥ 1 severe exacerbation in the past year
Retrospective, three 52-week, randomized, double-blind trials (TRINITY, TRILOGY, TRIBUTE) [45]	Definition 1: FEV_1_, SGRQ, exacerbations, death; Definition 2 (TRILOGY only): FEV_1_, SGRQ, exacerbations, TDI, death^a^	TRINITY: BDP/FORM/GLY vs TIO (CID 1 & 2) TRILOGY: BDP/FORM/GLY vs BDP/FORM (CID 1) TRIBUTE: BDP/FORM/GLY vs IND/GLY (CID 1)	High symptoms, at-risk population, CAT ≥ 10, FEV_1_ < 50% predicted plus ≥ 1 exacerbation in last year

Non-fatal CID worsening/suboptimal care in all trials was defined by changes from baseline in either: FEV_1_ ≥ 100 mL, SGRQ total score increase ≥ 4 units, CAT score increase ≥ 2 units, CCQ score increase ≥ 0.4 points; TDI focal score decrease of ≥ 1 unit; or a moderate/severe exacerbation

*ACL* aclidinium, *BDP* beclometasone dipropionate, *BUD* budesonide, *CAT* COPD Assessment Test, *CCQ* clinical COPD questionnaire, *CID* clinically important deterioration, *COPD* chronic obstructive pulmonary disorder, *FEV*_*1*_ forced expiratory volume in 1 s, *FF* fluticasone furoate, *FORM* formoterol fumarate, *GLY* glycopyrronium, *ICS* inhaled corticosteroid, *IND* indacaterol, *ITT* intent-to-treat, *LABA* long-acting β_2_-agonist, *LAMA* long-acting muscarinic receptor, *mMRC* modified Medical Research Council Dyspnea Scale, *SAL* salmeterol, *SFC* salmeterol/fluticasone, *SGRQ* St George’s respiratory questionnaire, *TDI* transition dyspnea index, *TIO* tiotropium, *UMEC* umeclidinium, *VI* vilanterol

^a^One analysis included death as a fatal CID event with no treatment impact

### Prognostic value of the composite CID endpoint

To determine the value of a composite CID endpoint, there are two key questions that need to be considered:

1. Does each individual CID component contribute to the composite?
2. Does the composite CID have prognostic ability?

To determine whether the composite CID endpoint meets each of these criteria, we have examined post hoc and a priori evidence to address each of these points.

#### Contribution of each individual CID component to the composite

**Fig. 2:**
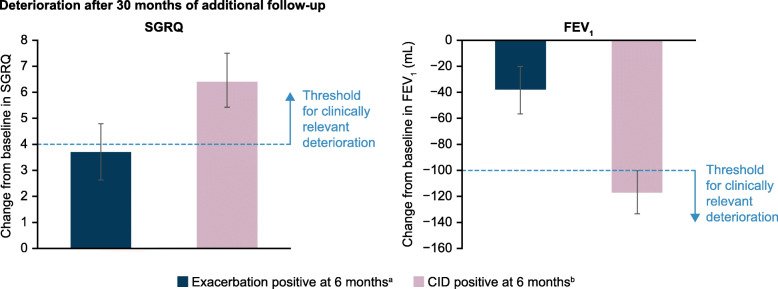
Exacerbations versus composite CID in measuring meaningful disease progression at 3-years in TORCH [48]. ^a^33% of the ITT were exacerbation positive in the first 6 months; ^b^54% of the ITT were CID positive in the first 6 months. CID, clinically important deterioration; FEV_1_, forced expiratory volume in 1 s; ITT, intent-to-treat; SGRQ, St George’s Respiratory Questionnaire

In a post hoc analysis of the 3-year TORCH clinical trial (which assessed mortality, exacerbations and health status with the ICS/long-acting β_2_-agonist (LABA) combination salmeterol/fluticasone propionate [SFC] versus its monotherapy components and placebo) [47], the composite CID endpoint assessed during the first 6 months predicted a clinically significant worsening of FEV_1_ and health status at 3 years. However, individual SGRQ worsening and the occurrence of an exacerbation (i.e. the non-FEV_1_ components of the CID) did not individually predict clinically relevant FEV_1_ deterioration [48]. Similarly, while the composite CID endpoint in the first 6 months predicted exacerbation risk and SGRQ worsening at 3 years, an exacerbation in the first 6 months only predicted future exacerbation risk. Overall, these findings demonstrate that each CID component in the first 6 months strongly predicted its own deterioration at 3 years, but on average did not predict a CID in another component. Therefore, individual CID components are the best predictors of their own deterioration, whereas the composite CID is a better holistic predictor of poor outcomes (whether these are a loss of lung function or health status, or the occurrence of an exacerbation) than any single CID component. For example, some exacerbations are unreported and are therefore untreated; untreated exacerbations have been shown to be associated with similar morbidity to treated exacerbations [33, 49, 50]. Consequently, relying on treated exacerbations alone as a prognositic indicator may underestimate the risk of disease progression by failing to detect all patients who are at risk of longer-term deterioration. This has been demonstrated in a post hoc analysis of the 3-year TORCH trial which compared deterioration using the composite CID or the exacerbation component alone during the first 6 months as a measure of potential clinically relevant disease progression at 3 years (Fig. 2) [48]. In TORCH, clinic visits were infrequent (every 6 months) thus opportunities to monitor health status and lung function changes were minimal. Nonetheless, this could reflect a real-world 6-month monitoring schedule. In a study in patients with an increased risk of exacerbations (due to a history of exacerbations in the previous year), there was minimal concordance between the different CID components, even though the probability of deterioration for each of the three CID components was significantly reduced by dual long-acting muscarinic antagonist (LAMA)/LABA bronchodilator therapy compared with ICS/LABA therapy [37]. This evidence confirms that individual CID component types are largely independent markers of treatment response and potential future deterioration, supporting the use of a composite measure.

#### Prognostic ability of a composite CID

**Fig. 3:**
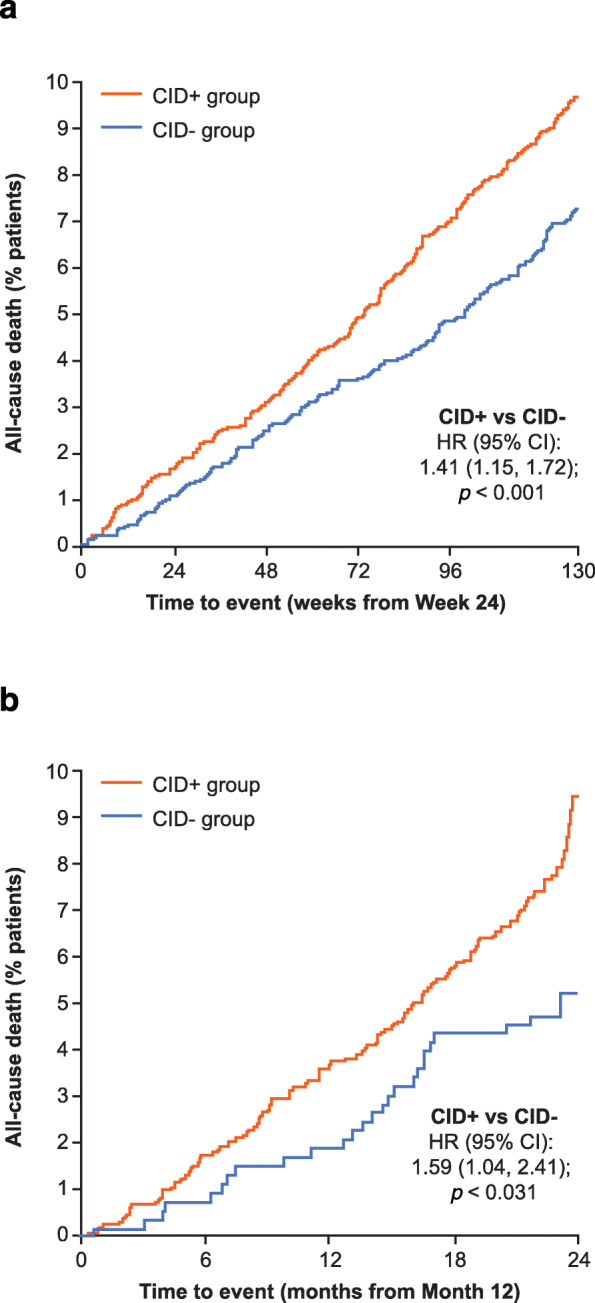
Time to all-cause mortality based on CID status in TORCH (**a**) and ECLIPSE (**b**)*.* Adapted from Naya I, et al Respir Res 2018. 19(1) p. 222.© The Authors 2018. Licensed under CC-BY 4.0 (http://creativecommons.org/licenses/by/4.0/). Post hoc analysis. Patients with any CID [CID+] compared with patients free of all CIDs [CID-]. ^a^At 6 months (CID+: *n* = 2870; CID-: *n* = 2422), ^b^at 12 months (CID+: *n* = 1442; CID-:531). CI, confidence interval; CID, clinically important deterioration; HR, hazard ratio

**Fig. 4:**
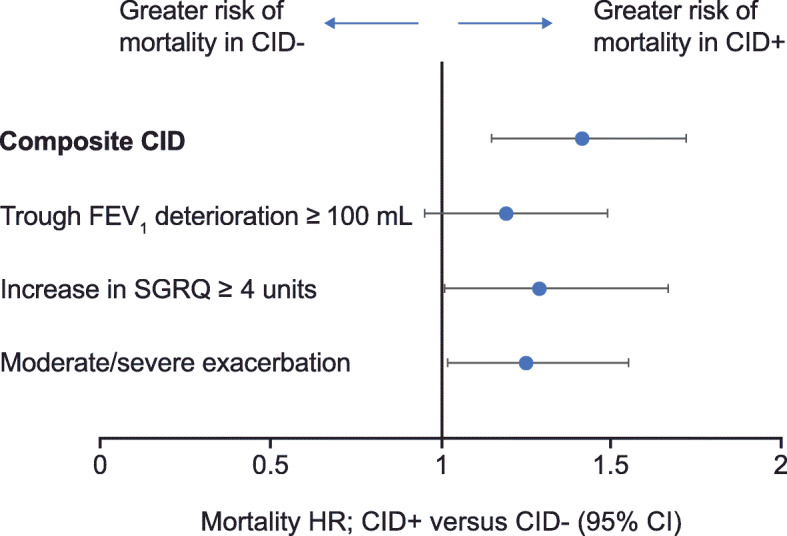
Prediction of all-cause mortality by CID status^a^ over 30 months of follow-up in TORCH [48]. ^a^CID status was assessed at 6 months. CI, confidence interval; CID, clinically important deterioration; CID+, cohort with a short-term deterioration (i.e. early unstable cohort); CID-, cohort without a short-term deterioration (i.e. early stable cohort); FEV_1_, forced expiratory volume in 1 s; HR, hazard ratio; SGRQ, St George’s Respiratory Questionnaire. Adapted from Naya I, et al. Respir Res 2018. 19(1) p. 222.© The Authors 2018. Licensed under CC-BY 4.0 (http://creativecommons.org/licenses/by/4.0/)

When evaluating a composite CID endpoint, it is important to determine whether the occurrence of a CID in the short-term can predict poorer long-term outcomes. Predicting an important outcome such as mortality constitutes powerful evidence of prognostic ability. A post hoc analysis of data from the TORCH and ECLIPSE studies showed an increased risk of all-cause mortality after a CID assessment at 6 and 12 months, respectively, in patients with any CID (CID+) compared with patients free of any CID (CID-) (Table 2 and Fig. 3) [48]. In the TORCH study, the CID+ subgroup compared with the CID- subgroup also had a significantly increased risk of exacerbations resulting in hospitalization after 6 months (Table 2) and a clinically meaningful loss of lung function and health status at 3 years (FEV_1_: − 117 mL; SGRQ: 6.42 units). In addition, the occurrence of a composite CID within the first 6 months predicted the risk of mortality to a greater extent than any single CID component. Each of the three CID components showed a broadly similar prognostic ability for mortality, although only SGRQ worsening and the occurrence of an exacerbation were statistically significant (Fig. 4). The CID+ subgroup (at 12 months) in the ECLIPSE study demonstrated an increased risk of mortality and an exacerbation resulting in hospitalization compared with the CID- subgroup during a 24-month follow-up (Table 2), as well as sustained deterioration in FEV_1_ and SGRQ score at 3 years (FEV_1_: − 115 mL; SGRQ: 4.67 units).

**Table 2:** Risk of long-term adverse outcomes by CID status in TORCH and ECLIPSE [48]

Outcome	TORCH (n = 5292)			ECLIPSE (n = 1953)		
	CID+ at 6 months [*N* = 2870], *n* (%)	CID- at 6 months [*N* = 2422], *n* (%)	% risk increase assessed at 7–36 months (95% CI)	CID+ at 12 months [*N* = 1442], *n* (%)	CID- at 12 months [*N* = 531], *n* (%)	% risk increase assessed at 13–36 months (95% CI)
Moderate/severe exacerbation	2082 (73)	1450 (60)	61 (50, 72)	1082 (75)	232 (44)	154 (120, 193)
Hospital admission for severe exacerbations	797 (28)	491 (20)	55 (38, 73)	454 (31)	66 (12)	181 (117, 263)
All-cause mortality	237 (8)	160 (7)	41 (15, 72)	121 (8)	27 (5)	59 (4, 141)

CID was defined as: FEV_1_, deterioration ≥ 100 mL or SGRQ deterioration ≥ 4 units or a first moderate/severe exacerbation on any treatment in both trials. All comparisons are for CID+ versus CID- cohorts. *p* < 0.05 for all risk increases in both trials

*CI* confidence interval, *CID* clinically important deterioration; *CID*+ cohort with a short-term deterioration (i.e. early unstable cohort); *CID*- cohort without a short-term deterioration (i.e. early stable cohort)

Results from a post hoc analysis of CID in the 1-year FULFIL [51] study, using the same composite definition, also demonstrated that a CID in the first 6 months (CID+ subgroup) was associated with a deterioration at 12 months in trough FEV_1_ and SGRQ score from baseline (FEV_1_: − 31 mL; SGRQ score: + 1.2 units) while the CID- subgroup showed marked clinical improvement from baseline (FEV_1_: + 169 mL; SGRQ score: − 10.1 units) [42]. In addition, a post hoc analysis of the FLAME study showed that patients with a CID at Week 12 had a significantly higher rate of moderate-to-severe exacerbations up to Week 52 (rate ratio: 1.8) [37]. The available evidence therefore suggests that the occurrence of a CID in the short term has prognostic ability with regard to multiple future outcomes.

### Ability of the composite CID to measure pharmacological treatment effects

One key application of the CID has been to compare the efficacy of pharmacological treatments in clinical trials. The populations of patients recruited to different trials may differ considerably with regard to their risk of future exacerbations, depending on the trial inclusion criteria. It is therefore important to address the question of whether the short-term CID endpoint can discriminate between different pharmacological treatments in patients at low risk (≤ 1 moderate exacerbation and no severe exacerbations in the previous year) and high risk (≥ 2 moderate exacerbations or ≥ 1 severe exacerbations in the previous year) of exacerbations [2]. Other relevant issues include whether different definitions for a composite CID endpoint improve the ability to measure treatment effects, and how study duration can influence CID results.

#### The composite CID in different patient populations

**Fig. 5:**
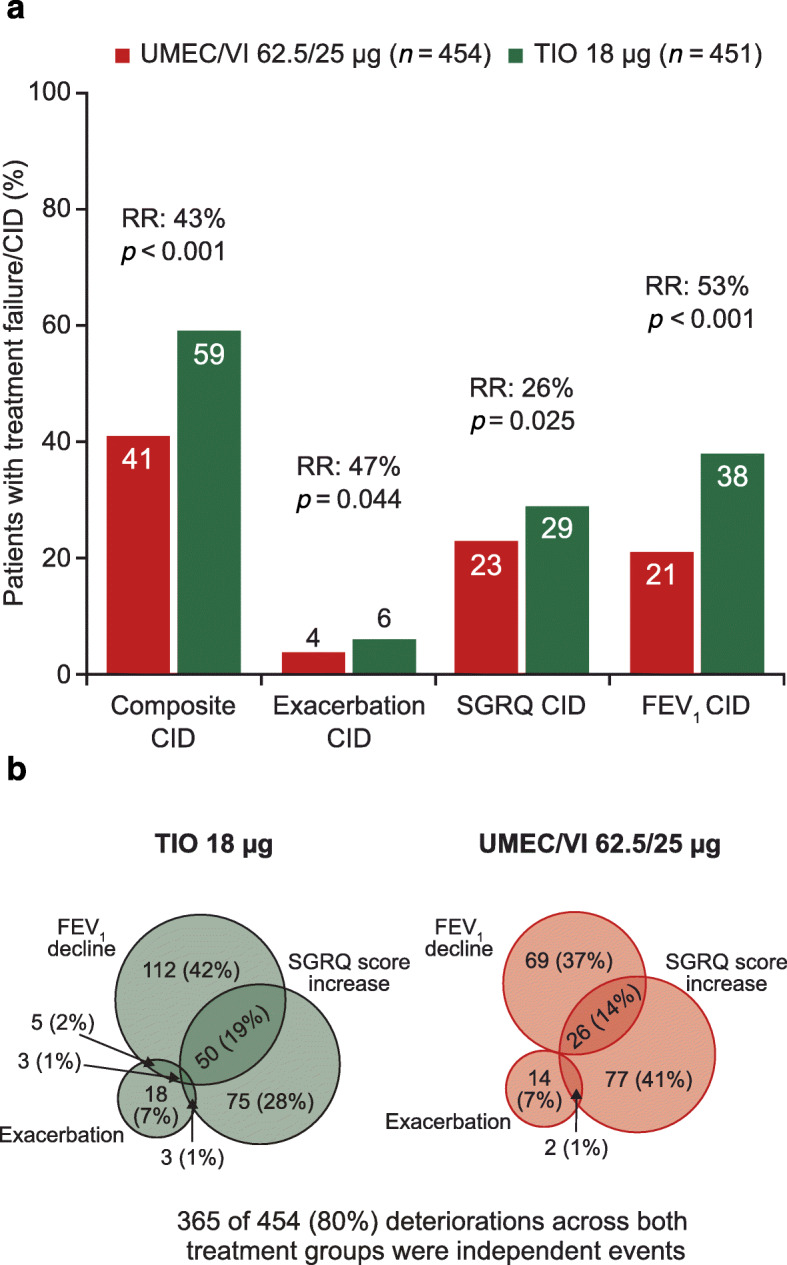
Incidence (**a**) and independence (**b**) of individual CID component types on bronchodilator therapy. **a** Adapted from Singh D, et al. Int J Chron Obstruct Pulmon Dis 2018. 19(1) p. 222.© The Authors 2016. Licensed under CC-BY 4.0 (http://creativecommons.org/licenses/by/4.0/). First CID was defined as a moderate/severe exacerbation and/or ≥ 100 mL decrease in trough FEV_1_ and/or ≥ 4 unit increase in SGRQ score; risk reduction was derived from time to first CID using a Cox’s proportional hazards model. CID, clinically important deterioration; FEV_1_, forced expiratory volume in 1 s; RR, risk reduction; SGRQ, St George’s respiratory questionnaire; TIO, tiotropium; UMEC/VI, umeclidinium/vilanterol

In a pooled post hoc analysis of two 24-week randomized clinical trials in symptomatic patients with a low risk of exacerbations [52, 53], at least 50% of patients receiving placebo or bronchodilator monotherapy experienced a CID (primarily due to lung function or health status deterioration) within 24 weeks [5]. However, the risk of a CID was significantly reduced in patients receiving dual bronchodilator therapy with umeclidinium/vilanterol (UMEC/VI) versus bronchodilator monotherapy (20 and 33% risk reduction versus UMEC and VI, respectively) or placebo (63%). In addition, UMEC/VI significantly reduced all three CID components compared with tiotropium (TIO), with a 43% risk reduction overall (Fig. 5a). In most patients who experienced a CID, this was due to worsening on a single CID component, with two or more deteriorations occurring in only 20% of CID+ patients (Fig. 5b). Another post hoc analysis of three 26-week, randomized clinical trials in patients with COPD at a low risk of exacerbations demonstrated that dual bronchodilation with indacaterol/glycopyrronium (IND/GLY) significantly reduced the risk of a first CID versus either TIO (incidence of first CID: 47% vs 59%; hazard ratio [HR] = 0.72) or the ICS/LABA combination SFC (incidence of first CID: 38% vs 50%; HR = 0.67) [38]. Similarly, a post hoc analysis of three 24-week randomized studies comparing UMEC/VI and TIO in symptomatic patients with low exacerbation risk, UMEC/VI significantly reduced the risk of short-term CID versus TIO by 38 and 34% in the intent-to-treat and maintenance-treatment-naïve populations, respectively [41]. In contrast to the analyses described above, which were performed post hoc, the recent EMAX study, performed in a symptomatic low exacerbation risk population who were not receiving ICS, prospectively evaluated CID risk and showed that UMEC/VI significantly reduced the risk of a first CID compared with UMEC (17%) and salmeterol (38%) [46].

Fewer studies have used the composite CID endpoint to compare pharmacological therapies in patients at high risk of exacerbations. However, a post hoc analysis of the FLAME study [54] in patients with a history of ≥ 1 exacerbation in the previous year demonstrated that IND/GLY significantly delayed the time to CID compared with SFC [37]. The risk of a CID was reduced by 28%, and the risk of worsening in all three CID components was also significantly reduced with IND/GLY versus SFC. In another post hoc analysis of data from three large 12-month studies (TRILOGY, TRINITY, and TRIBUTE) in patients with symptomatic COPD and at increased risk of exacerbations, single-inhaler triple therapy with beclometasone dipropionate (BDP)/formoterol fumarate (FORM)/GLY significantly reduced the incidence of CID compared with LAMA (TIO) monotherapy (TIO;74% vs 82%; HR = 0.72), ICS/LABA (BDP/FORM; 70% vs 84%; HR = 0.61), and LAMA/LABA (IND/GLY; CID incidence: 82% vs 87%; HR = 0.82) [45]. Taken together, these findings suggest that treatment effects on CID risk can be used to discriminate between pharmacological therapies in patients at high and low risk of future exacerbations.

Findings from these studies also suggest that the contribution of exacerbations to the overall rate of CID events is increased in populations at high risk of exacerbations. Among patients at low risk of exacerbations, the proportion of patients with an on-treatment exacerbation contributing to a CID has been reported to be between 6 and 14% across numerous 24-week trials [5, 41, 44]. Among patients at high risk of future exacerbations, 44–50% of the randomized patients in the FLAME study had an on-treatment exacerbation [37, 42] while 31–38% had an exacerbation in any treatment arm in the TRILOGY, TRINITY, and TRIBUTE studies [45]. These findings suggest that exacerbations make a greater contribution to CID risk in patients at higher risk for exacerbations than in those with lower risk. However, it should be noted that the FLAME, TRILOGY, TRINITY, and TRIBUTE studies in high-risk populations were longer than those in low-risk populations (52 weeks versus 24 weeks), and in the 24-week FULFIL study the proportion of high-risk patients with an on-treatment exacerbation was only 10–14% [42]. Further analyses of the contribution of exacerbations to the CID in populations of different exacerbation risks is warranted.

#### Comparisons of alternative composite CID endpoint definitions

The original composite CID definition uses the SGRQ to assess health status; however, this is a complex and time-consuming measure, and a composite definition that uses a simpler measure of health status such as CAT could therefore be advantageous. A CAT-based CID definition provided comparable results to the original SGRQ-based definition in prospective analyses in the FULFIL and EMAX studies [42, 46]. In addition, some studies have analyzed composite CID endpoints that included TDI [38, 45]. In post hoc analyses of the TRILOGY study, the deteriorations identified by TDI (BDP/FORM/GLY: 25%; BDP/FORM: 30%) were fewer than those identified by SGRQ (BDP/FORM/GLY: 41%; BDP/FORM: 47%); however, the hazard ratio for a first deterioration with BDP/FORM/GLY compared with BDP/FORM was very similar for the two measures (SGRQ: 0.78; TDI: 0.81) [45]. Similarly, including TDI to make a 4-component composite CID endpoint slightly increased the number of patients with a CID (by 3–4% in each treatment group), but did not alter the treatment difference. The EMAX study also prospectively assessed CID risk using the original 3-component SGRQ-based definition as well as a 4-component CID definition including SGRQ, TDI, CAT, and a moderate/severe exacerbation [46]. An increased treatment benefit with UMEC/VI compared with either UMEC or SAL was demonstrated regardless of whether a FEV_1_ component was included in the CID composite, and there was little change in the treatment difference observed with the 4-component definition compared with either of the 3-component SGRQ- and CAT-based definitions [46]. These findings indicate that FEV_1_ is not a dominant driver of the risk of deterioration and that there is little advantage to adding a second PRO to the composite. Furthermore, there are practical problems associated with TDI since it relies on remembering the baseline state (unlike SGRQ), which becomes more distant and problematic in longer studies.

Although there are differences in CID definitions, all of those described include components that the GOLD strategy recommends monitoring in order to assess treatment response [2]. Severe adverse events, such as cardiovascular events and pneumonia, could potentially be included in the CID composite; indeed death has been included in some analyses [45]. However, although such events are clinically important, they are rare in clinical trials in COPD and for a composite endpoint to be a useful measure it is important that the components occur at a similar incidence and have a similar impact on patient health [55]. Furthermore, the principal strength of the composite CID is its ability to predict future outcomes such as hospital admissions and death [48], which is not feasible for patients who have died. In our opinion, the inclusion of death as a component of the CID is therefore of little value, although we acknowledge that it is important to evaluate the incidence of adverse events in addition to CID when assessing patient response to treatment and the benefit:risk ratio.

Some studies have used a variation on the composite CID endpoint known as the sustained CID, which describes a worsening that was maintained across multiple subsequent study visits. When used as a supportive secondary assessment in several studies, the overall number of patients with a CID was approximately halved but the treatment differences remained unaltered [5, 41, 44]. However, this method has not been evaluated in terms of prognostic ability; consequently, its value in monitoring deterioration is uncertain.

#### Treatment comparisons using CID in studies of different durations

Many of the studies discussed so far have demonstrated treatment differences in CID risk over 24–26 weeks duration [5, 38, 41, 44, 46]. Reductions in the risk of a first CID with LAMA/LABA versus ICS/LABA [38] and triple therapy versus ICS/LABA, LAMA/LABA, or LAMA monotherapy [45] have also been demonstrated over 52 weeks. In addition, a prospective CID analysis in the FULFIL study demonstrated that once-daily single inhaler triple therapy with fluticasone furoate (FF)/UMEC/VI significantly reduced the risk of a first CID by 51–52% over 24 weeks and 47–48% over 52 weeks compared with twice-daily budesonide (BUD)/FORM depending whether the original CID definition or a CAT-based CID definition was used [42].

A potential advantage of using the CID to compare novel and existing pharmacological treatments is that it may allow studies of shorter duration than would be possible using individual endpoints to assess clinically meaningful improvements. In support of this, CID has been used to compare pharmacological therapies in shorter studies. A post hoc analysis of four 12-week studies tested the benefit of escalation to triple therapy among patients receiving ICS/LABA or dual bronchodilator therapy in symptomatic patients at high risk of exacerbation or hospitalization. All patients received an ICS/LABA at study entry, and in patients randomized to receive a LAMA (UMEC) in addition, the risk of a CID was 45–58% lower than in those who received ICS/LABA [43]. A post hoc analysis of the randomized, open-label CRYSTAL trial also showed a significant 59% reduction in the risk of a first CID (using a definition based on FEV_1_, TDI, and moderate/severe exacerbations) during a 12-week treatment period after switching from LAMA or LABA monotherapy to IND/GLY, compared with patients who did not switch [40].

These analyses therefore suggest that the CID endpoint can be used to evaluate differences between treatments within relatively short 12-week studies. For treatments with novel mechanisms of action (beyond bronchodilators and ICS) in clinical development, use of the CID endpoint has the potential to provide evidence of clinical efficacy without the need to conduct studies of a long duration.

### Use of CID in clinical practice

COPD is a heterogeneous and complex disease. The multidimensional CID endpoint, which incorporates aspects of lung function, health status and exacerbations, captures different aspects of deterioration or disease activity in COPD whilst having the potential to be a simple tool for use in clinical practice. It may therefore assist physicians by providing a holistic evaluation of patients’ health. Its prognostic ability will also enable the identification of individuals who may benefit from earlier intensification of treatment. For example, it may identify high-risk patients and/or those who would benefit from additional treatment, but who may have previously been considered to have low risk based on FEV_1_ or the absence of exacerbations. Identification of such patients earlier in the course of their disease could enable intensification of treatment and potentially reduce the risk of disease worsening. However, it should be noted that if the CID was to be used as a prognostic tool to identify patients at risk, it would be important to compare the CID with existing well-established prognostic tools such as BODE.

There are some limitations that should be considered if CID is to be implemented as a routine tool in clinical practice. The majority of CID analyses to date have used pre-bronchodilator FEV_1_ to detect objective worsening in lung function, but due to the time-consuming nature of diagnostic spirometry, alternative approaches to assess meaningful deterioration are required to enable efficient clinical assessment of CID status. One possible alternative that should be evaluated in future studies is the use of small handheld microspirometry devices to measure FEV_1_/FEV_6_ ratio, where FEV_6_ is defined as FEV in 6 s, a surrogate for forced vital capacity [56]. Furthermore, the SGRQ is too complex and time consuming for routine use, but the available evidence suggests that the CAT could be substituted as the measure of health status. Further studies assessing the feasibility of replacing the SGRQ with the CAT in the CID would be an important step toward facilitating implementation of the tool in a clinical setting, particularly in primary care.

### Suggested areas for future application

The studies discussed here have used the CID as an outcome measure to show that escalation of therapy can reduce the risk of a CID in a broad spectrum of patients [5, 37, 38, 41–43, 52, 53, 57]. However, no study to date has directly assessed the potential benefit of stepping-up treatment in response to a CID (ie, using the CID as a trigger for treatment intensification). The extent to which a short-term CID can be reversed, with a consequent reduction in the risk of future events, is a key topic for further investigation. For example, consideration should be given to studies in which the CID is used as a monitoring tool to identify high-risk patients who have milder airflow limitation and may currently warrant only short-acting bronchodilator therapy, to determine whether earlier intensification of maintenance treatment can alter their disease progression. The prognostic ability of the CID may also allow for a reduction in the size or length of a clinical trial in which it is included as an endpoint. In addition, real-world evidence studies of the CID are currently lacking and would be a valuable addition to the field. Finally, exploration of alternative cut-off points for deterioration may help to improve understanding of the relationship between CID thresholds and long-term outcomes, allowing for improved personalization of care.

## Conclusions

**Fig. 6:**
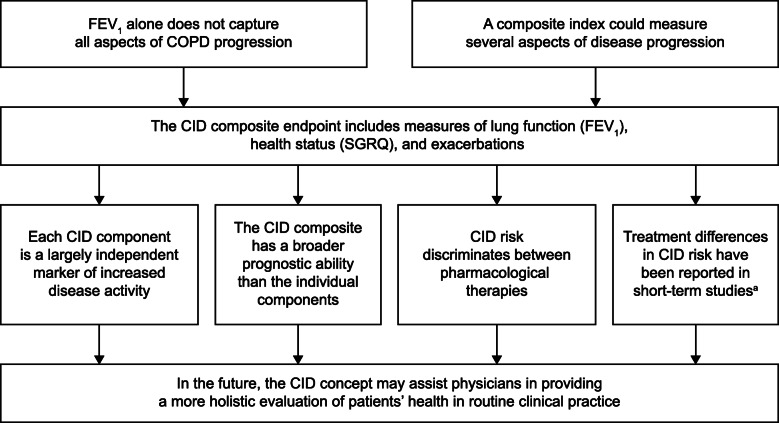
Summary of the CID concept. ^a^within 12 to 24 weeks. CID, clinically important deterioration; COPD, chronic obstructive pulmonary disease; FEV_1_, forced expiratory volume in 1 s; SGRQ, St George’s Respiratory Questionnaire

There is a widely acknowledged need for an individualized holistic approach to the management of COPD. To help meet that need, the composite CID endpoint captures key features of the complex and heterogeneous nature of COPD, including lung function, health status and exacerbations, to assess disease activity and progression (Fig. 6). Each component acts as a largely independent marker of suboptimal treatment response, making a unique contribution to the composite endpoint. Short-term CID has demonstrated prognostic value in the prediction of poor long-term outcomes and can be used in clinical trials to differentiate pharmacological treatments in patients at high and low risk of future exacerbations. CID is a promising concept that has the potential to facilitate better treatment and management decisions for patients with COPD.

## Data Availability

Not applicable.

## Abbreviations

ACL: aclidinium
ADO: Age, dyspnea, obstruction
BODE: Body-mass index, airflow obstruction, dyspnea, and exercise
BDP: Beclometasone dipropionate
BUD: Budesonide
CAT: COPD assessment test
CART: Classification and regression tree
CCQ: Clinical COPD Questionnaire
CI: confidence interval
CID: Clinically important deterioration
COPD: Chronic obstructive pulmonary disease
DOSE: Dyspnea, obstruction, smoking, exacerbation
FEV_1_: Forced expiratory volume in 1 s
FEV_6_: Forced expiratory volume in 6 s
FF: Fluticasone furoate
FORM: Formoterol fumarate
GLY: Glycopyrronium
GOLD: Global Initiative for Chronic Obstructive Lung Disease
HADO: Health, activity, dyspnea, obstruction
HR: Hazard ratio
ICS: Inhaled corticosteroids
IND: Indacaterol
ITT: intent-to-treat
LABA: Long-acting β_2_-agonist
LAMA: Long-acting muscarinic antagonist
MCID: Minimal clinically important difference
mMRC: modified Medical Research Council
PRO: patient-reported outcome
RR: risk reduction
SAFE: SGRQ score, airflow limitation and exercise tolerance
SAL: salmeterol
SFC: Salmeterol/fluticasone propionate
SGRQ: St George’s respiratory questionnaire
TIO: Tiotropium
TDI: Transition Dyspnea Index
UMEC: Umeclidinium
VI: Vilanterol
