# Correction to: Measuring disease activity in COPD: is clinically important deterioration the answer?

**DOI:** 10.1186/s12931-021-01894-7

**Published:** 2021-11-20

**Authors:** Dave Singh, Gerard J. Criner, Ian Naya, Paul W. Jones, Lee Tombs, David A. Lipson, MeiLan K. Han

**Affiliations:** 1 Medicines Evaluation Unit, Manchester University NHS Foundation Trust, University of Manchester, Manchester, UK; 2 Lewis Katz School of Medicine, Temple University, Philadelphia, PA USA; 3 GSK, Respiratory Medicines Development Centre, Stockley Park, Middlesex UK; 4 RAMAX Ltd, Bramhall, Cheshire UK; 5 Precise Approach Ltd, London, UK; 6 GSK, Respiratory Clinical Sciences, Collegeville, PA USA; 7 Division of Pulmonary, Allergy, and Critical Care, Perelman School of Medicine, University of Pennsylvania, Philadelphia, PA USA; 8 Division of Pulmonary and Critical Care, University of Michigan Health System, Ann Arbor, MI USA

## Correction to: Respir Res (2020) 21:134 10.1186/s12931-020-01387-z

The original version of the article unfortunately contained a mistake in the disclosure statement.

It has been corrected in this correction.

It should read as,

Disclosure section

The disclosure statement was incomplete, the correct statement is “MeiLan K Han received writing and research support from GlaxoSmithKline for this work. Outside this work, she also reports personal fees from GlaxoSmithKline, Boehringer Ingelheim, AstraZeneca, Merck and Mylan. She also reports research support from Novartis and Sunovion.”
